# Clinical and radiological features of intracranial ancient schwannomas: a single-institution, retrospective analysis

**DOI:** 10.1007/s10014-024-00482-z

**Published:** 2024-04-05

**Authors:** Takahiro Tsuchiya, Masako Ikemura, Satoru Miyawaki, Yu Teranishi, Kenta Ohara, Tetsuo Ushiku, Nobuhito Saito

**Affiliations:** 1 Department of Neurosurgery, Faculty of Medicine, The University of Tokyo, 7-3-1 Hongo, Bunkyo-ku, Tokyo, 113-8655 Japan; 2 Department of Pathology, Faculty of Medicine, The University of Tokyo, 7-3-1 Hongo, Bunkyo-ku, Tokyo, Japan

**Keywords:** Ancient schwannoma, Intracranial schwannoma, Cyst

## Abstract

Ancient schwannoma (AS) is a subtype of schwannoma characterized by slow progression despite degenerative changes in pathology. Although it is considered a benign tumor, most previous reports have focused on extracranial AS; therefore, the clinical characteristics of intracranial AS is not clear. We included 174 patients who underwent surgery for sporadic intracranial schwannoma, and 13 patients (7.5%) were diagnosed with AS. Cysts were significantly more common in patients with AS than conventional schwannomas (92.3% vs. 44.7%, p < 0.001), as was bleeding (38.5% vs. 6.9%, p = 0.003) and calcification (15.4% vs. 1.3%, p = 0.029). The maximum tumor diameter was also larger in patients with AS (35 mm vs. 29 mm, p = 0.017). The median duration from symptom onset to surgery (7.0 vs. 12.5 months, p = 0.740) did not significantly differ between groups, nor did the probability of postoperative recurrence (p = 0.949). Intracranial AS was strongly associated with cyst formation and exhibited a benign clinical course with a lower rate of recurrence and need for salvage treatment. Extracranial AS is reportedly characterized by a slow progression through a long-term clinical course, whereas intracranial AS did not progress slowly in our study and exhibited different clinical features to those reported for extracranial AS.

## Introduction

Schwannomas are benign nerve sheath tumors composed of differentiated, neoplastic Schwann cells. Schwannomas are classified into various subtypes, including conventional, cellular, plexiform, epithelioid, and melanotic schwannomas [29, 39]. Ancient schwannoma (AS) is another subtype, first described by Ackerman et al. in 1951 [1]. It is characterized by pathological nuclear atypia without a fission pattern or degenerative changes, such as hemorrhage, cyst formation, and hyalinization [1, 7, 21]. AS is a benign tumor (World Health Organization [WHO] tumors of the central nervous system [CNS] grade 1) with a low Ki-67 index (2–3%) [36], and malignancy is rare. Immunostaining of this tumor is positive for S-100, similar to that for other schwannoma subtypes [15, 25, 28, 34]. Since diagnosis is difficult based only on clinical and radiological features [36], pathological diagnosis is important [21]. Clinically, AS is characterized by a slow progression through a long-term clinical course [16], and pathological results of degenerative changes are thought to support this clinical feature.

Previous reports included the proportion of AS compared to all schwannomas, but all of those reports concerned extracranial schwannomas, and the proportion varied widely (3.2–78.9%) [1, 3, 5, 15, 43]. AS is often reported to occur extracranially in the retroperitoneum, head, and neck [14, 30, 37]. On the other hand, reports focused on intracranial AS are rare. To our knowledge, only seven cases of intracranial AS have been reported, all in case reports [2, 3, 22, 25, 32, 34, 35]. Therefore, the clinical and radiological features of intracranial AS remain unknown. Although intracranial AS is considered benign owing to the features of extracranial AS, the validity of such an inference is not confirmed.

We previously reported on a case of rapid progression and early postoperative recurrence of intracranial AS [34]. In this study, we aimed to clarify the clinical and radiological features of intracranial AS.

## Methods

### Patient data

**Fig. 1:**
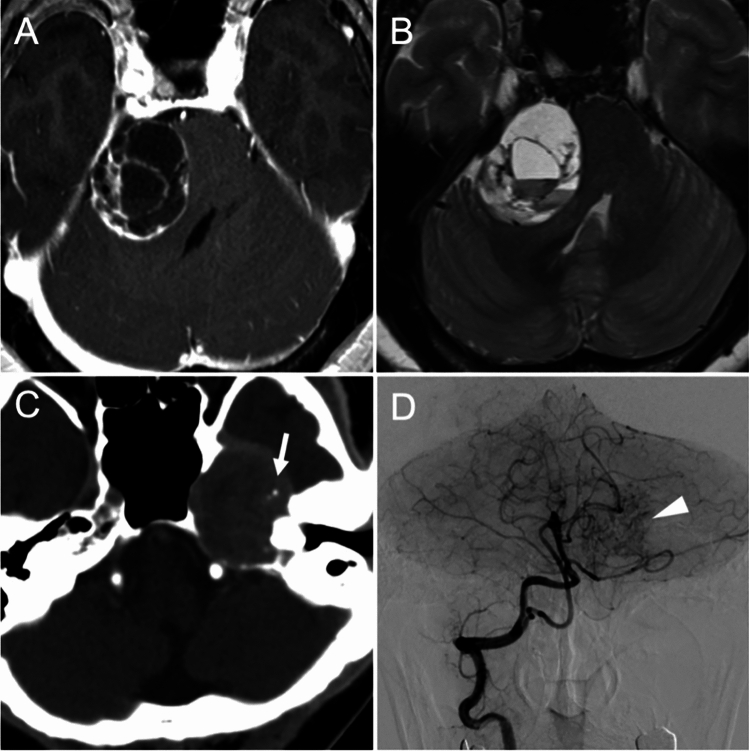
Preoperative imaging studies for AS. Gadolinium-enhanced T1-weighted MRI revealed cyst formation (**A**), and niveau formation was observed upon T2-weighted MRI, indicative of intratumoral hemorrhage (**B**). Calcification was observed as a high-density area upon CT (**C**, arrow). Angiography was performed in some of the cases to evaluate the presence of tumor staining (**D**, arrowhead)

A total of 181 consecutive patients underwent surgery for sporadic intracranial schwannomas at our hospital from February 2000 to March 2021. Among them, 7 were excluded because they underwent surgery for recurrent tumors, and 174 were included in the analysis. For all 174 cases, clinical information and radiological features were retrospectively collected via chart review. Preoperative computed tomography (CT) and magnetic resonance imaging (MRI) were performed in all cases to evaluate the presence of cysts, bleeding, and calcification (Fig. 1A–C). Preoperative angiography was performed in 106 cases to evaluate the presence of tumor staining with contrast material, which is a characteristic of hypervascular schwannomas (Fig. 1D) [33]. Complete tumor removal was designated gross total resection (GTR), and anything less than complete resection was designated subtotal resection. Salvage treatment (surgery or radiosurgery) had been performed for clinical symptoms or regrowth of the residual tumor of ≥ 2 mm by the last follow-up MRI compared with the residual tumor size at the previous postoperative imaging. This single-institution, retrospective cohort study was approved by the ethics committee of The University of Tokyo Hospital (approval number: 2231). Written informed consent was obtained from all the patients.

### Histopathology and immunohistochemistry

Hematoxylin- and eosin-stained and immunohistochemical slides were reviewed for all cases by experienced neuropathologists. AS was defined as tissue with conspicuous nuclear atypia with degenerative changes such as hemorrhage with hemosiderin deposition, hyalinized blood vessels, and lymphocytic infiltration, according to the fifth edition of the WHO classification and previous reports [12, 19–21, 28, 39]. While some degree of partial nuclear atypia or degenerative changes are often observed in conventional schwannomas, the diagnosis of AS was made when such findings are diffuse or multiple locations throughout the tumor. Ki-67 staining was performed on all schwannomas to calculate the Ki-67 index.

### Statistical analysis

Univariate analysis was conducted using Fisher’s exact test for categorical variables and the Mann–Whitney U test for continuous variables. Thereafter, multivariable analysis was conducted using logistic regression for variables that exhibited significant differences in the univariate analyses. In addition, patients were grouped into two categories based on the maximum tumor size (smaller [< 30 mm] vs. larger [≥ 30 mm]). These variables were incorporated as explanatory variables in the multivariable analysis. The median durations from symptom onset to surgery and the final postoperative follow-up were calculated for all patients. The duration from initial surgery to recurrence was calculated using the Kaplan–Meier method and compared using the log-rank test. Statistical tests were performed using R software (ver. 4.0.2; The R Project for Statistical Computing, Vienna, Austria), and statistical significance was set at a p value < 0.05.

## Results

### Clinical characteristics

The clinical characteristics of the 174 patients are presented in Table 1. The median age at the time of surgery was 43 years, and 84 patients (48.3%) were men. Overall, 132 tumors were vestibular schwannomas (75.9%) and 42 were nonvestibular schwannomas (24.1%). Concurrent of meningiomas were observed in four patients (2.3%). The median value of the maximum tumor diameter was 30 mm. From the preoperative CT and MRI evaluations, cysts, bleeding, and calcifications were discovered in 84 (48.3%), 17 (9.8%), and 4 (2.3%) patients, respectively. GTR was performed in 130 (74.7%) patients. The median time from symptom onset to surgery was 12.0 months. Salvage treatment, such as surgery or radiosurgery, was performed in 13 patients (7.5%). Postoperative recurrence was observed in 14 patients (8.0%).

**Table 1:** Clinicopathological characteristics of 174 schwannomas

Characteristics (N = 174)	Number
Age at surgery, years, median (range)	43 (14–75)
Male (%)	84 (48.3)
Lesion site (%)	
Vestibular nerve	132 (75.9)
Trigeminal nerve	16 (9.2)
Lower cranial nerve	21 (12.1)
Others	5 (2.9)
Laterality (%)	
Left	84 (48.3)
Right	89 (51.1)
Meningioma (%)	4 (2.3)
Maximum tumor diameter, mm, median (range)	30 (8–102)
≥ 30 mm (%)	88 (50.6)
Cyst (%)	84 (48.3)
Bleeding (%)	17 (9.8)
Calcification (%)	4 (2.3)
Tumor stain (%)	25 (14.4)
Gross total resection (%)	130 (74.7)
Pathology (%)	
Conventional schwannoma	159 (91.4)
Ancient schwannoma	13 (7.5)
Cellular schwannoma	2 (1.1)
Ki-67 index (mean ± SD)	2.2 ± 3.3
Symptom to surgery, months, median (range)	12.0 (2.0–144.0)
Postoperative follow-up, months, median (range)	61.4 (0.7–222.6)
Salvage treatment (%)	13 (7.5)
Postoperative recurrence (%)	14 (8.0)

*SD* standard deviation

### Histopathological features

**Fig. 2:**
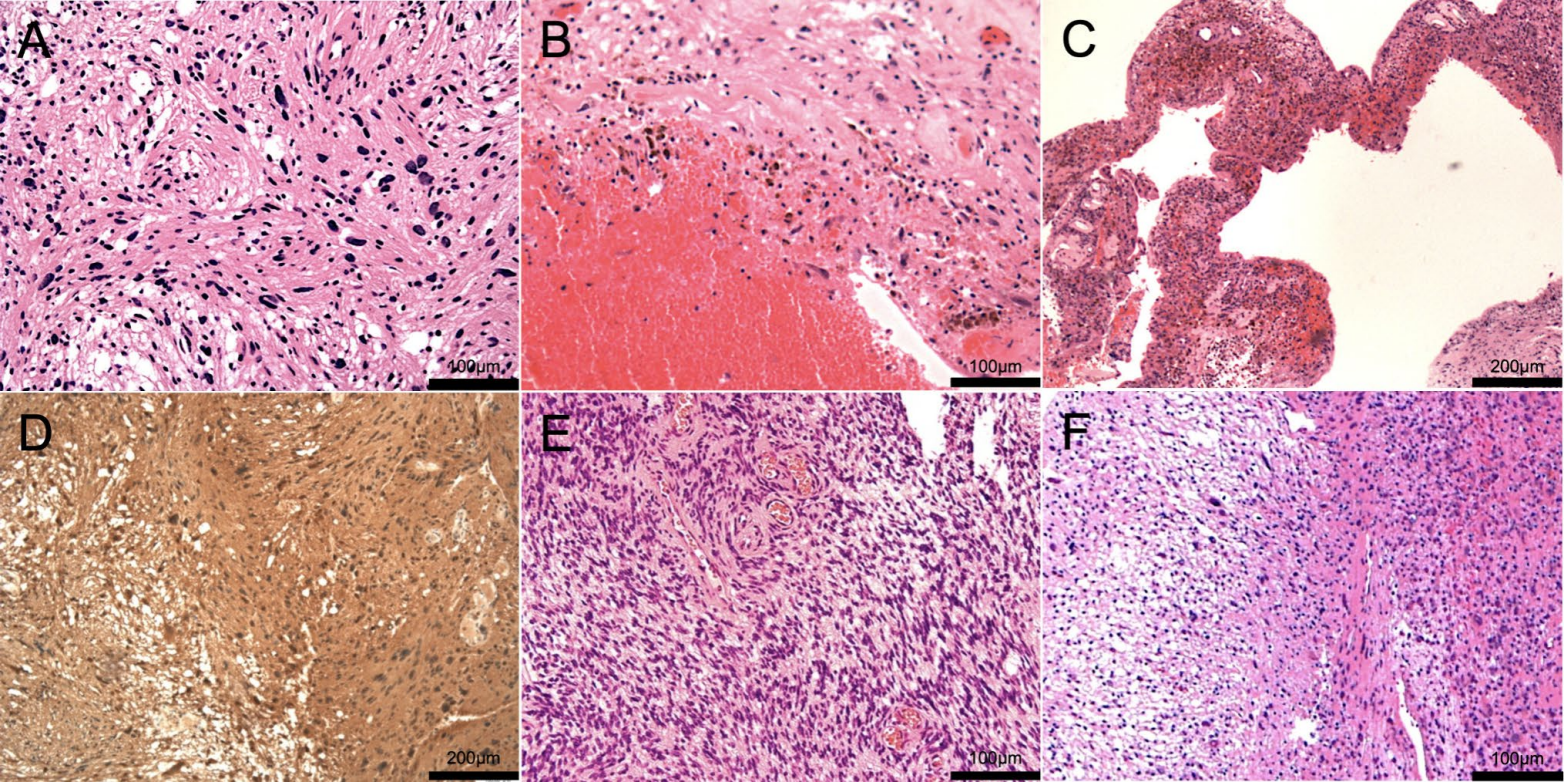
Histopathological features of AS and other subtypes of schwannomas. Hematoxylin–eosin staining of AS demonstrated nuclear atypia without mitosis (**A**, original magnification: ×200) in addition to degenerative changes, such as hemorrhage with hemosiderin deposition (**B**, original magnification: × 200), hyalinized blood vessels, and cyst formation (**C**, original magnification: ×100). Immunostaining of AS revealed diffuse S100 positivity (**D**, original magnification: ×100), which was also seen in all types of schwannomas. Hematoxylin–eosin staining of cellular schwannomas demonstrated increased cellularity with a lack of Verocay bodies (**E**, original magnification: ×200). Hematoxylin–eosin staining of conventional schwannomas exhibited cellular areas alternating with hypocellular areas (**F**, original magnification: ×200)

Of the 174 patients, 13 (7.5%) were diagnosed with AS, 2 (1.1%) with cellular schwannomas, and the others with conventional schwannomas. AS exhibited nuclear atypia without mitosis in addition to degenerative changes, such as hemorrhage with hemosiderin deposition, hyalinized blood vessels, and cyst formation (Fig. 2A–C). Immunostaining of AS revealed diffuse S100 positivity, which was also seen in all types of schwannomas (Fig. 2D). Cellular schwannomas exhibited increased cellularity with a lack of Verocay bodies (Fig. 2E). Two cases of cellular schwannoma were observed in our cohort. One of them was relatively malignant, with a Ki-67 index of > 30% in some areas, but the mitosis was not evident overall, and most of the areas had no Ki-67 positive tumor cells, with diffuse S100 positivity, so the diagnosis was cellular schwannoma rather than malignant peripheral nerve sheath tumor. Conventional schwannomas exhibited cellular areas alternating with hypocellular area (Fig. 2F). The average Ki-67 index for all of the schwannomas was 2.2%. The Ki-67 index was significantly associated with the need for salvage treatment (p = 0.046). In contrast, although the Ki-67 index seemed positively related to postoperative recurrence, this relationship was not statistically significant (p = 0.098).

### Comparison of conventional schwannoma and AS

Among 159 patients with conventional schwannomas, 120 had vestibular schwannomas (75.5%) and 39 had non-vestibular schwannomas (24.5%). Moreover, among the 13 patients with AS, 11 had vestibular schwannomas (84.6%) and 2 had non-vestibular schwannomas (15.4%). The frequency of vestibular schwannomas did not differ between patients with conventional schwannomas and those with AS (p = 0.736). Univariate analysis of conventional schwannoma and AS revealed that patients with AS had a significantly larger maximum tumor diameter (35 mm vs. 29 mm, p = 0.017) and occurrence of cysts (92.3% vs. 44.7%, p < 0.001), bleeding (38.5% vs. 6.9%, p = 0.003), and calcification (15.4% vs. 1.3%, p = 0.029) upon preoperative imaging (Table 2). The median duration from symptom onset to surgery (7.0 vs. 12.5 months, p = 0.740), salvage treatment rate (7.7% vs. 6.9%, p > 0.99), and postoperative recurrence rate (7.7% vs. 7.5%, p > 0.99) did not significantly differ between patients with AS and those with conventional schwannomas. The probability of postoperative recurrence did not significantly differ between patients with conventional schwannomas and those with AS (p = 0.949). The Ki-67 indices of patients with conventional schwannomas and those with AS also did not significantly differ (mean ± standard deviation = 1.6 ± 1.2% vs. 2.1 ± 2.6%, p = 0.578). Multivariable analysis was conducted using the four variables (tumor size, cysts, bleeding, and calcification) that significantly differed between groups in the univariate analyses (Table 3); cysts and calcification were associated with AS rather than with conventional schwannomas (p = 0.034 and p = 0.048, respectively).

**Table 2:** Univariate analysis of conventional schwannoma and ancient schwannoma

Variable	Conventional schwannoma (N = 159)	AS (N = 13)	Cellular schwannoma (N = 2)	p value*
Age, years, median (range)	43 (15–75)	44 (14–73)	31 (24–37)	0.522
Male (%)	76 (47.8%)	6 (46.2%)	1 (50.0%)	> 0.99
Meningioma (%)	3 (1.9%)	1 (7.7%)	0 (0.0%)	0.272
Maximum tumor diameter, mm, median (range)	29 (8–102)	35 (25–60)	38 (32–44)	**0.017**
Cyst (%)	71 (44.7%)	12 (92.3%)	1 (50.0%)	**< 0.001**
Bleeding (%)	11 (6.9%)	5 (38.5%)	1 (50.0%)	**0.003**
Calcification (%)	2 (1.3%)	2 (15.4%)	0 (0.0%)	**0.029**
Tumor stain (%)	22 (13.8%)	2 (15.4%)	1 (50.0%)	> 0.99
Gross total resection (%)	118 (74.2%)	11 (84.6%)	1 (50.0%)	0.522
Ki-67 index (mean ± SD)	2.1 ± 2.6	1.6 ± 1.2	15.5 ± 14.5	0.578
Symptom onset to surgery, months, median (range)	12.5 (2.0–144.0)	7.0 (3.0–120.0)	3 (3–3)	0.740
Salvage treatment (%)	11 (6.9%)	1 (7.7%)	1 (50.0%)	> 0.99
Postoperative recurrence (%)	12 (7.5%)	1 (7.7%)	1 (50.0%)	> 0.99

Bold indicates *p* < 0.05

*AS* ancient schwannoma

*Conventional schwannoma vs. AS

**Table 3:** Factors associated with ancient schwannoma

Variable	Odds ratio	95%CI	p value
Maximum tumor diameter^a^	1.58	0.41–6.06	0.506
Cyst	10.00	1.19–84.60	**0.034**
Bleeding	3.72	0.90–15.30	0.070
Calcification	11.10	1.02–120.00	**0.048**

Bold indicates *p* < 0.05

^a^Maximum tumor diameter of 30 mm or more

## Discussion

### Radiological features of intracranial AS

This study revealed that AS is characterized by a larger tumor diameter, prevalence of cysts, prevalence of bleeding, and prevalence of calcification than conventional schwannomas. AS in the head and neck are reportedly large, which is consistent with that in this study [14]. One of the other radiological features we analyzed was tumor staining upon angiography, characteristic of hypervascular vestibular schwannoma (HVS) [33]. In our study, 14.4% of the patients had HVS, which was not associated with AS. Few reports on calcification in patients with schwannomas have been published. Zhang et al. reported that blood supply may be related to calcification, but no consensus has been reached [42]. AS are reported to exhibit hemosiderin deposition upon pathologic analysis [13, 26], and the higher prevalence of bleeding in our cohort supports this feature. Furthermore, additional evaluation at T2-star-weighted MRI would have accurately assessed hemosiderin deposition.

Multivariable analysis revealed that cysts were a significant feature of patients with AS. In our cohort, the cysts varied from single to multi-cystic, regardless of the schwannoma subtype, and in some cases the cysts accounted for the majority of the tumor volume. Intracranial cystic schwannomas are reportedly large [13, 17, 24, 41], and have a short clinical history owing to rapid growth [24, 31, 34]. In this study, the presence of cysts was significantly associated with AS; 12/13 (92.3%) AS cases were cystic AS. In the seven previous reports of intracranial AS, five mentioned the presence of cysts (71.4%), which confirms our results [2, 3, 22, 25, 32, 35]. Therefore, AS may be a subtype of cystic schwannomas. However, cystic schwannomas are characterized by rapid growth and a short clinical history. This feature is inconsistent with AS, which is traditionally characterized by slow progression. In our study, the time from symptom onset to surgery among patients with intracranial AS actually seemed shorter than that among patients with conventional schwannomas, although this result was not statistically significant. In some reports, cases of AS have exhibited rapid progression [34]. To explain this seeming discrepancy, intracranial and extracranial AS may have fundamentally different clinical features. This idea is supported by a report by Ando et al. that cysts are more common in intracranial schwannomas than in extracranial schwannomas [4].

### Histopathological features

AS is difficult to diagnose based on clinical and radiological features alone; hence, definitive pathological diagnosis is important [36]. Pathological features of AS include nuclear atypia without mitosis or degenerative changes. Immunostaining of AS is positive for S-100 and SOX10, as for other schwannomas [15, 28, 39]. Although quantitative evaluation has not been performed, AS cases in our cohort were notable for hemosiderin deposition, hyalinized vessels, and inflammatory cell infiltration, in addition to nuclear atypia. The Ki-67 index is low and consistent with WHO CNS grade 1 [39]. Recent research demonstrates that the Ki-67 index is closely associated with tumor recurrence, especially in cases with a Ki-67 index of 3% or higher [27]. In our cohort, the Ki-67 index was associated with salvage treatment, supporting the results of previous reports. Furthermore, in our patients with AS cases, the mean Ki-67 index was 1.6%, which is a pathologically favorable histology similar to that of conventional schwannomas. Malignancy or sarcoma may be misdiagnosed in patients with extracranial AS because of the presence of nuclear atypia upon pathological evaluation [11, 18, 21]. Mikami et al. reported on a patient with cervical AS with malignant transformation [23], in which the Ki-67 index was quite high (30.5%), suggesting that the Ki-67 index can support the diagnosis of benign or malignant disease. In addition, there was one AS case that developed an early postoperative recurrence requiring stereotactic radiosurgery. In this case, the Ki-67 index was 5%, which is relatively high compared to the overall average of 1.6% and may have been associated with the risk of postoperative recurrence [27].

In our cohort, two patients had cellular schwannomas (1.1%). Cellular schwannomas reportedly account for 4.6–6.7% of all schwannomas [8–10], and the proportion of cellular schwannomas within our cohort was small compared to that in previous reports. One of the two patients with a cellular schwannoma in this study had a short time from symptom onset to surgery (3 months), and their Ki-67 index was partially > 30%. D'Almeida et al. reported the clinicopathological features of 20 patients with cellular schwannomas [9]. In that study, the Ki-67 index in patients with cellular schwannomas was higher than that of patients with conventional schwannomas, and cellular schwannomas exhibited locally aggressive growth, consistent with our two cases. The lack of sufficient cases and data regarding cellular schwannomas in our study impeded us from further characterizing this subtype.

### Comparison of intracranial and extracranial AS

Isobe et al. reported an average of 8.3 years from symptom onset to surgery among seven patients with extracranial AS, concluding that extracranial AS is a slow-growing tumor [6, 16, 40]. The long-term progression of the tumor is thought to produce degenerative changes, which is why the tumor is termed "ancient.” In contrast, in our study, the time from symptom onset to surgery did not significantly differ between AS and conventional schwannoma. This difference can be explained by the characteristics of the tumor extension sites. Extracranial lesions, such as those in the retroperitoneal cavity, may reach a substantial size by the time of diagnosis because of the non-restrictive and expandable properties of the retroperitoneal space [38]. In contrast, the intracranial space is surrounded by the skull, and the space in the skull base where most schwannomas occur is especially narrow. Therefore, intracranial AS often presents with neurological symptoms at an early stage of development, leading to prompt diagnosis and treatment.

These results raise the question of whether the pathology of intracranial AS is truly the result of an “ancient” process. Rather, intracranial AS was significantly associated with cyst formation in this study, which is consistent with the results of seven previous case reports. Thus, the features of intracranial AS seem to differ from those of extracranial AS.

### Limitations

The data used in this study were retrospectively collected. Although the sample size was considered adequate for the analysis of AS, only two cases of cellular schwannomas were included. A larger sample would allow for more detailed subtype analysis. In addition, the lack of objective criteria in the WHO classification for the evaluation of degenerative findings in AS has prevented quantitative evaluation. Furthermore, genetic analysis, which was not performed in this study, may clarify the diagnostic criteria and improve our understanding of the pathogenesis of this disease.

## Conclusions

Herein, we presented the first report of the prevalence of AS among patients with intracranial schwannomas and its clinical and radiological features in a single-institution, retrospective study. Intracranial AS was strongly associated with cyst formation and exhibited a benign clinical course with postoperative recurrence and infrequent need for salvage treatment; however, it did not exhibit slow progression. Intracranial AS seems to present clinical features different from those of extracranial AS.

## Data Availability

The data that support the findings of this study are available from the corresponding author on reasonable request.
